# Evaluation of Phenolic Phytochemical Enriched Commercial Plant Extracts on the *In Vitro* Inhibition of α-Glucosidase

**DOI:** 10.3389/fnut.2017.00056

**Published:** 2017-11-10

**Authors:** Allie Brown, Danielle Anderson, Kenneth Racicot, Sarah J. Pilkenton, Emmanouil Apostolidis

**Affiliations:** ^1^Department of Chemistry and Food Science, Framingham State University, Framingham, MA, United States; ^2^Combat Feeding Directorate, Natick Solider Research, Development, and Engineering Center (NSRDEC), Natick, MA, United States

**Keywords:** glucose management, phenolic phytochemicals, antioxidants, glucosidase inhibition, cranberry extract

## Abstract

Green tea (GT), cranberry (CR), and tart cherry extracts were evaluated for their ability to inhibit yeast α-glucosidase, relevant to glucose uptake. The total phenolic content (TPC), antioxidant activity, and *in vitro* inhibitory activity of yeast α-glucosidase were examined for the extracts in the present study. GT had higher TPC and antioxidant activity, but CR demonstrated a greater α-glucosidase inhibitory activity, on phenolic basis. CR was fractionated using LH-20 column chromatography into two fractions: 30% methanol (CME) and 70% acetone (CAE). TPC, antioxidant activity, and yeast α-glucosidase inhibitory activity were determined for the fractions. CAE had a greater TPC and antioxidant activity than CME, but the two fractions had a synergistic effect when inhibiting yeast α-glucosidase. Our findings suggest that CR has the greatest potential to possibly manage post-prandial blood glucose levels *via* the inhibition of α-glucosidase, and that the effect is through synergistic activity of the extract’s phenolic compounds.

## Introduction

Type 2 diabetes mellitus (T2DM) is a metabolic disease in which the body does not properly use insulin (1). Individuals with T2DM develop hyperglycemia due to their inability to secrete insulin or use insulin properly (1). As of 2014, 29.1 million people in the United States have diabetes, with T2DM accounting for over 27 million cases (2, 3). Diabetes can lead to other metabolic diseases such as cardiovascular disease and can cause damage, dysfunction, or failure of various organs, including the heart, kidney, eyes, nerves, and blood vessels (1, 4).

Prediabetes is a risk state that defines the high chance of developing diabetes, where blood glucose levels are above normal, but below the diabetes threshold (5). According to the American Diabetes Association, about 70% of individuals with prediabetes will eventually develop diabetes (3). The prevention of prediabetes would inhibit individuals from developing diabetes and other complications that are associated with the disease. A possible intervention involves the inhibition the carbohydrate hydrolyzing enzymes that metabolize starch molecules to glucose molecules.

Dietary carbohydrates are broken down by salivary and pancreatic α-amylase to oligosaccharides (6, 7). The oligo- and disaccharides are further broken down to single glucose molecules by α-glucosidases in the small intestine (1, 6, 7). The inhibition of α-glucosidases prevents oligosaccharides from being broken down to glucose, resulting to reduced levels of glucose to be absorbed from the small intestine to the blood.

Acarbose is considered a last-line intervention treatment for T2DM (8). The two sugar units within the structure of the compound form the functional inhibitory site that binds to α-glucosidases in the small intestine and α-amylase in the pancreas (9). Due to the mechanistic nature of acarbose, patients complained of gastrointestinal discomfort, flatulence, abdominal cramps, nausea, and diarrhea when using acarbose and consuming a high carbohydrate diet (8).

The buildup of glucose in T2DM patients can be inhibited by using phenolic phytochemical compounds from plants through the inhibition of carbohydrate hydrolyzing enzymes, namely α-glucosidases. Various research papers have suggested the inhibitory effect of phenolic phytochemicals on carbohydrate hydrolyzing enzymes, but without the excessive side effects observed with acarbose. Examples include cinnamon (*Cinnamomum zeylanicum B.)* and black tea polyphenols that exhibited strong inhibition of α-glucosidase *in vitro* (10, 11).

Phenolic phytochemicals are produced within plants through the shikimate and/or acetate–malonate pathways and are metabolized by various pathways in order to obtain their final chemical structure, which is based on their biological function (12). There are more than 8,000 phenolic compounds, which are distributed into classes based on the number of phenolic rings in the structure and the constituents that bind to the rings (13). Phenolic compounds are further categorized as flavonoids and non-flavonoids (14). The sub groups within the flavonoid category include flavonols, flavones, isoflavones, flavanones, anthocyanidins, and flavanols (13). The flavanols subclass includes catechins and proanthocyanidins (PACs) (13).

Phenolic phytochemicals have various benefits, including antioxidant activity, reduce inflammation, modulate detoxifying enzymes, stimulate the immune system, regulate gene expression in cell proliferation and apoptosis, and participate in hormone metabolism (15–18). It is believed that the phenolic compounds in food products work synergistically with one another. In oral cancer cell lines, KB and CAL-27, the total phenolic fraction of cranberry (CR) extract had a greater inhibition of proliferation when compared to the anthocyanin and PAC fractions (19). The same trend was observed in colon cancer cell lines (HT-29, HCT116, SW480, and SW620) and prostate cancer cell lines (RWPE-1, RWPE-2, and 22Rv1) (19). The combination of ellagic acid (ELA) and grape seed extract (GSE) rich in PACs and resveratrol caused a significant reduction in Bcl-2 mRNA in SENCAR mice with 7,12-dimethylbenz[a]anthracene-induced skin carcinoma when compared to GSE and ELA alone (20). Due to the synergistic effect of phenolic phytochemicals, it is not uncommon to observe reduction of bioactivity while performing fractionation-guided bioactivity assays.

Phenolic phytochemicals are documented inhibitors of α-glucosidase and could reduce or retard glucose uptake in the small intestine, *via* inhibition of disaccharide digestion. This property could be potentially used to reduce the prevalence of type 2 diabetes but can also be an effective mediator for energy uptake for individuals who need to consume high-energy diets, such as soldiers. The aim of this investigation was to evaluate the commercially available powdered extracts, tart cherry (TC), green tea (GT), and CR, which are currently being evaluated for use in military rations. Initially, all extracts were evaluated for their total phenolic contents (TPCs), antioxidant activities, and α-glucosidase inhibitory activities. Then the most bioactive extract, in terms of α-glucosidase inhibitory activity, was further fractionated and evaluated to possibly identify its bioactive components.

## Materials and Methods

### Materials

The GT extract powder was provided by Pharmachem Laboratories, Inc. (Anaheim, CA, USA) with a product number of 8GRE21200. The TC extract powder was a VitaCherry^®^ Tart Cherry RapiDry™ Powder, product number N298, from FutureCeuticals (Momence, IL, USA). The CR extract powder was provided by Ocean Spray Cranberries, Inc. (Lakeville, MA, USA). Sigma-Aldrich provided the chemicals used in the experiments, which includes Folin–Ciocalteu reagent, 2,2-diphenyl-1-picrylhydrazyl (DPPH) reagent, ethanol, 4-nitrophenyl α-D-glucopyranoside (pNPG), dimethyl sulfoxide (DMSO), yeast α-glucosidase (*Saccharomyces cerevisiae*), acetone, methanol, and HPLC grade phosphoric acid and acetonitrile (St. Louis, MO, USA).

### LH-20 Column Fractionation

The CR extract powder was subjected to LH-20 (Sigma Lipophilic Saphead, Sigma-Aldrich, St. Louis, MO, USA) column extraction to initially separate the low and high molecular weight phenolic phytochemicals. For the CR stock solution, 50.0 g of CR extract powder was dissolved in 300 mL of distilled water. Five milliliters of CR stock solution was added to the 40 mL LH-20 column. The tube of the column was connected to an ISCO Tris pump (Teledyne Technologies International Corporation, Thousand Oaks, CA, USA). The pump was turned to speed 30 in the forward direction and the 10 × mode. The pump was turned off once the sample was fully in the column. The column was fully washed with approximately 600 mL of 30% methanol. The pump was turned to speed 50 and the fraction was collected once the effluent was red and until it was very light pink. The column was then washed with approximately 400 mL of 70% acetone and the fraction was collected immediately after CME was collected and until the elution was very light purple. The fractions were evaporated using a Büchi rotary evaporator R-144 (Cole Parmer, Vernon Hills, IL, USA) at 50°C until the final volume of 200 mL was achieved. The concentrated fractions were transferred to 600 mL freeze drying vials and placed in the −60°C Queue Cryostar blast freezer (Westbury, NY, USA) for 24 h. The fractions were freeze dried using a VirTis Automatic Freeze Dryer (Marshall Scientific, Hampton, NH, USA). The fractions were freeze dried for about a week, or until the sample was dry. The powder was weighed and stored in a plastic vial at −18°C.

### TPC Determination

Stock solutions of the phytochemical extract powders were prepared by dissolving 1.0 g of the phytochemical extract powder in 50.0 mL distilled water. A 50× and 100× dilution was made for CR and GT, respectively. TC did not require a dilution. For CME, 10.0 mg powder was dissolved in 10.0 mL distilled water. For CAE, 10.0 mg powder was dissolved in 10.0 mL 50% DMSO. Both fractions were diluted 2×. The method described by Kang et al. (21) was followed. For the standard curve preparation, gallic acid was dissolved in ethanol at various concentrations (0, 10, 50, 100, 150, and 250 µg gallic acid/mL ethanol). The total soluble phenolic content was expressed as mg/g gallic acid equivalents (GAE).

### Antioxidant Activity Evaluation

The antioxidant activity of the phytochemical extract powders and the CR fractions was identified using the DPPH Assay. The stock DPPH solution was diluted with 95% ethanol to obtain an absorbance value between 1.8 and 2.0 at 517 nm. The blank used in the assay was 95% ethanol. Briefly, in a cuvette, 0.4 mL of the sample was added along with 2.0 mL of diluted DPPH solution. After 5 min, the absorbance was measured at 517 nm using a Lambda 11 UV/Vis spectrometer (Perkin Elmer, Waltham, MA, USA). The TC samples were centrifuged (to remove interfering additives) using an AccuSpin Micro 17 R centrifuge at 13,200 rpm and 24°C (Thermo Fisher Scientific, Waltham, MA, USA) during the 5-min reaction time. The stock solutions of each extract from the TPC assay were used. For GT, 500, 1000, and 5000× dilutions were analyzed. For CR, 50, 100, and 150× dilutions were analyzed. For TC, 0 and 2× dilutions were analyzed. For the CR fractions, CME was diluted 25, 50, and 100 × and CAE was diluted 100, 250, and 500×. The control for the whole extract powders and CME was 0.4 mL distilled water and 0.4 mL 50% DMSO for CAE. The antioxidant activity (%) was calculated (Eq. 1). The IC_50_ value, expressed as milligram per milliliter solids, was calculated for each phytochemical extract powder using the equation for the line for the graph of antioxidant activity (%) versus concentration of the sample (milligram per milliliter solids) (Eq. 2). The *y* variable was equal to 50 for the IC_50_ calculation.

(1) $$\text{Antioxidant activity }\left(\%\right)=(({\text{Abs}}_{\text{control}}-{\text{Abs}}_{\text{sample}})/{\text{Abs}}_{\text{control}})\times 100.$$

(2) $${\text{IC}}_{x}\text{ value}=e^{\left((y\;-\;b)/m\right)}.$$

### Yeast α-Glucosidase Inhibitory Activity Evaluation

The extract powders and CR fractions were evaluated for their inhibiting activity against yeast α-glucosidase. For the phytochemical powders, 10.0 mg of powder was dissolved in 10.0 mL of distilled water. The GT extract powder was diluted 50, 100, 250, and 500×. The CR extract powder was diluted 2, 5, 10, and 20×. The TC extract power was diluted 0, 2, and 5×. The TC powder was centrifuged for 5 min at 13,200 rpm and 24°C to remove interfering additives using an AccuSpin Micro 17 R centrifuge (Thermo Fisher Scientific, Waltham, MA, USA) before dilutions were made. For CME, 10.0 mg powder was dissolved in 10.0 mL of distilled water. For CAE, 10.0 mg powder was dissolved in 10.0 mL of 50% DMSO. CME was diluted 2, 5, and 10× and CAE was diluted 2, 5, 10, and 20×.

Following dilutions, 50 µL of sample to be tested were transferred in the wells of a 96 well plate. For the control, distilled water was added instead of sample. To each well with sample or blank, 50 µL 0.1 M phosphate buffer was added followed by 100 µL *Saccharomyces cerevisiae* enzyme solution (10 U/mL). The well plate was incubated at room temperature for 10 min. The sample was placed in the BioTek EC ×800UV microplate reader (Winooski, VT, USA) and the method was set up during the incubation time using the Gen 5, version 2.9 software. After incubation, 50 µL 5 mM pNPG was added to each well except for the blank wells. In the blank wells, 50 µL 0.1 M phosphate buffer was added. The absorbance of the samples was measured at 405 nm every minute over a 5-min period.

The yeast α-glucosidase inhibition activity (%Inhibition) was calculated using the blank value and the 5-min values of the control and the sample (Eq. 3). The IC_50_ value was calculated for each extract powder and the IC_20_ value was calculated for CME and CAE using the equation for the line for the graph of %Inhibition versus TPC (μg/mL) (Eq. 2). The *y* variable was equal to 50 for the IC_50_ calculation and 20 for the IC_20_ calculation.

(3) $$\%\text{ Inhibition}=\left[\frac{\left(({\text{Sample}}_{5\min}-{\text{Sample}}_{\text{Blank}})-({\text{Control}}_{5\min}-{\text{Control}}_{\text{Blank}})\right)}{\left({\text{Sample}}_{5\min}-{\text{Sample}}_{\text{Blank}}\right)}\right]\times 100.$$

### HPLC Phenolic Profiling

The whole CR extract powder and the methanol and acetone fractions were analyzed using HPLC to determine the phenolic phytochemical composition of each sample. The stock solutions used for total soluble phenolic content were standardized to contain 1 mg/g phenols/1 mL water. The solutions were placed in 1.5 mL Eppendorf tubes and centrifuged for 5 min at 13,200 rpm and 24°C using an AccuSpin Micro 17 R centrifuge (Thermo Fisher Scientific, Waltham, MA, USA). The supernatant of each sample was collected into HPLC vials and stored at 4°C until analysis.

The instrument used was a Finnigan Surveyor HPLC equipped with an LC Pump Plus quaternary pump, an autosampler, and a PDA Plus detector operating on XCalibur software (Thermo Fisher Scientific, Waltham, MA, USA). At the time of the analysis, the samples were placed in the autosampler, which was thermostated at 4°C. The injection volume of the samples was 5 µL. The column used was a Waters Symmetry Shield RP 18 with dimensions of 4.6 × 250 mm and 5 µm particle size (Waters Corporation, Milford, MA, USA). The column was held at 35°C for the course of the analysis. The method used was a modification of the method used by Seeram et al. (19). Briefly, the mobile phase consisted of 4% phosphoric acid (solvent A) and acetonitrile (solvent B). Linear gradient elution conditions were used at a flow rate of 0.75 mL/min, starting with 95% solvent A and being decreased to 65% solvent A over 81 min. The phenolic phytochemical compounds were observed using a UV-Vis photodiode array detector at wavelengths of 254, 280, 320, 360, and 520 nm and a total scan between 200 and 600 nm. The HPLC data was graphed comparing the time of elution and the intensity of the peak. The λ_max_ values of the major peaks were identified.

### Statistical Analysis

All experiments were performed three times in triplicates. Means, SDs, and IC_50_/IC_20_ values (concentrations resulting to 50 or 20% inhibition) were calculated from replicates within the experiments and analyses using Microsoft Excel XP. The significance of each group was verified with one-way analysis of variance.

## Results

### Total Phenolic Content

**Figure 1:**
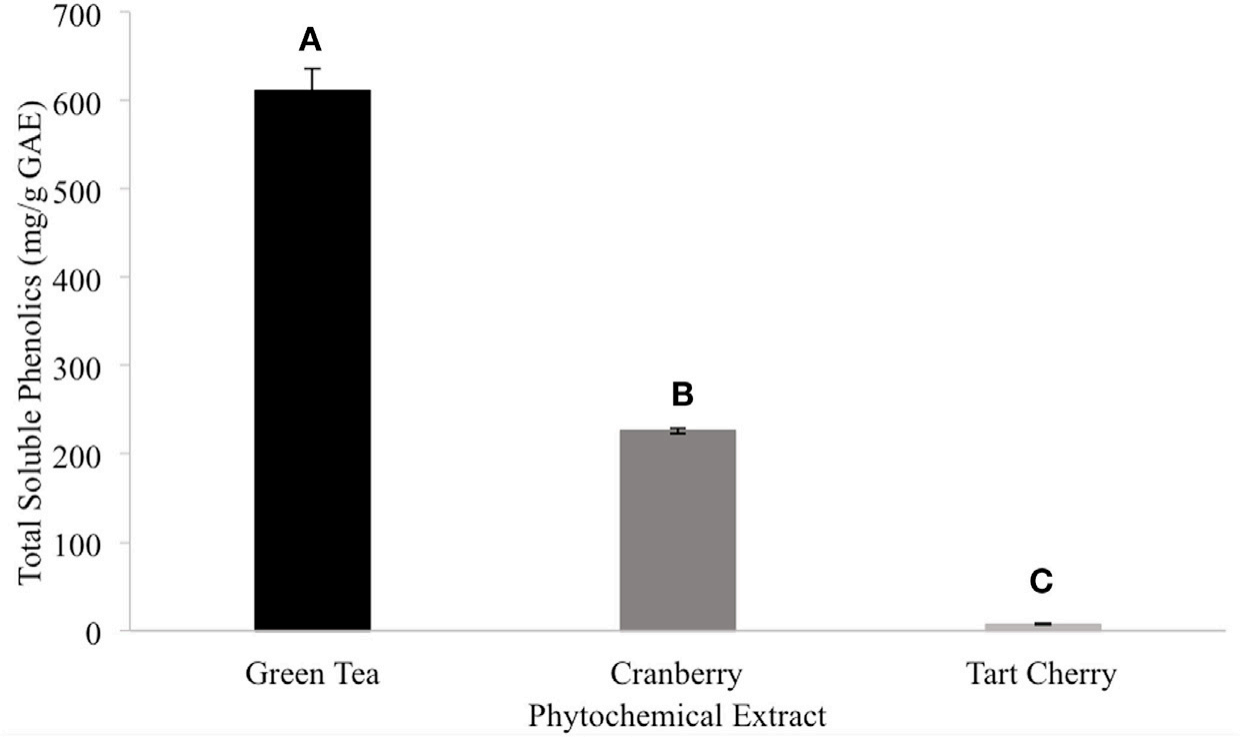
The total phenolic content of the extract powders (different letters signify significant different values, *p* < 0.05).

**Figure 2:**
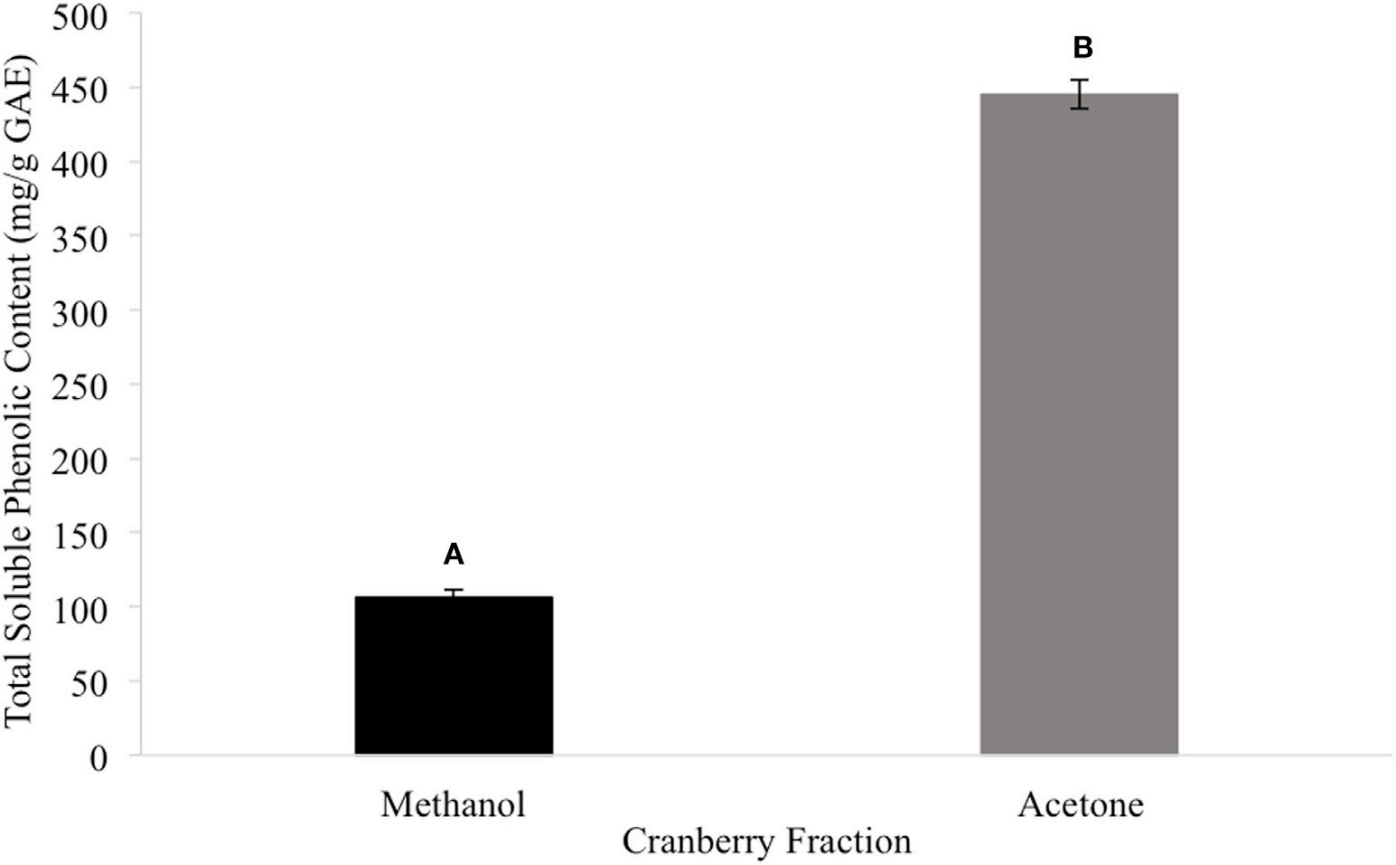
The total phenolic content of the cranberry fractions (different letters signify significant different values, *p* < 0.05).

Among the tested extract powders, the GT extract powder had the highest TPC with a value of 628.03 ± 12.31 mg/g GAE (Figure 1). The CR extract powder had the second highest TPC with a value of 278.02 ± 4.37 mg/g GAE (Figure 1). The TC extract powder had the lowest TPC with a value of 5.29 ± 0.092 mg/g GAE (Figure 1). Among the tested CR fractions, the methanolic fraction (CME) had a lower TPC than the acetone fraction (CAE), with values of 106.29 and 445.17 mg/g GAE, respectively (Figure 2).

### Antioxidant Activity

The observed antioxidant activities correlated to the observed phenolic contents. More specifically, the GT powder had greater antioxidant activity and TC had the lowest. The IC_50_ values for GT, CR, and TC were 0.039, 0.26, and 14.67 mg/mL solids, respectively (Table 1). Among the tested CR fractions, CAE had greater antioxidant activity than CME. The IC_50_ values for CME and CAE were 0.54 and 0.068 mg/mL solids, respectively (Table 2). It is important to also note that CAE also had greater antioxidant activity than the whole CR extract powder (Tables 1 and 2).

**Table 1:** The IC_50_ values for the antioxidant activity of the extract powders.

Extract Powder	IC_50_ (mg/mL solids)
Green tea	0.039
Cranberry	0.26
Tart cherry	14.67

**Table 2:** The IC_50_ values for the antioxidant activity of the cranberry fractions.

Fraction	IC_50_ (mg/mL solids)
Methanol	0.54
Acetone	0.068

### Yeast α-Glucosidase Inhibitory Activity

All tested samples had a dose-dependent inhibitory activity against alpha-glucosidase. To understand the correlation between the observed inhibitory activities and the different phenolic profiles of the extracts, we expressed our observations as IC_50_ phenolic basis (phenolic content needed for 50% inhibition). Based on the IC_50_ values, the CR extract powder had better yeast α-glucosidase inhibitory activity than the GT or TC extract powders. More specifically, the IC_50_ values for the GT, CR, and TC extract powders were 68.98, 20.34, and ?105.80 μg/mL TPC, respectively (Table 3). These observations suggest that the phenolic compounds in the CR extract powder have higher *in vitro* inhibitory activity against α-glucosidase.

**Table 3:** The IC_50_ values for the yeast α-glucosidase inhibitory activity of the extract powders (tart cherry did not result to inhibition greater than 50% at any tested dose).

Extract Powder	IC_50_ (μg/mL Total Phenolic Content)
Green tea	68.98
Cranberry	20.34
Tart cherry	>105.80

Since the phenolic compounds in the CR extract had higher bioactivity, we decided to fractionate the CR extract (as described in the Section “Materials and Methods”) and to evaluate which fraction is responsible for the observed effect. The resulting fractions had reduced inhibitory activity when compared to the whole extract, and no fraction resulted to inhibition above 50% (at the tested doses). For this reason, our observations were expressed as IC_20_ phenolic basis (phenolic content for 20% inhibition) for comparison purposes. The IC_20_ values were used to compare the two fractions to each other and to the whole CR extract powder. CME had a lower IC_20_ value than CAE, with values of 51.29 and 101.78 µg/mL TPC, respectively (Table 4). The corresponding whole CR IC_20_ value was 6.12 µg/mL TPC (Table 4). Our observations suggest that the observed CR extract alpha-glucosidase inhibitory activity is due to the synergistic effect of the two resulting fractions.

**Table 4:** The IC_20_ values for the yeast α-glucosidase inhibitory activity of the whole cranberry (CR) extract and CR fractions.

Sample	IC_20_ (μg/mL Total Phenolic Content)
Whole CR	6.12
Methanol fraction (CME)	51.29
Acetone fraction (CAE)	101.78

### HPLC Phenolic Profiling

**Figure 3:**
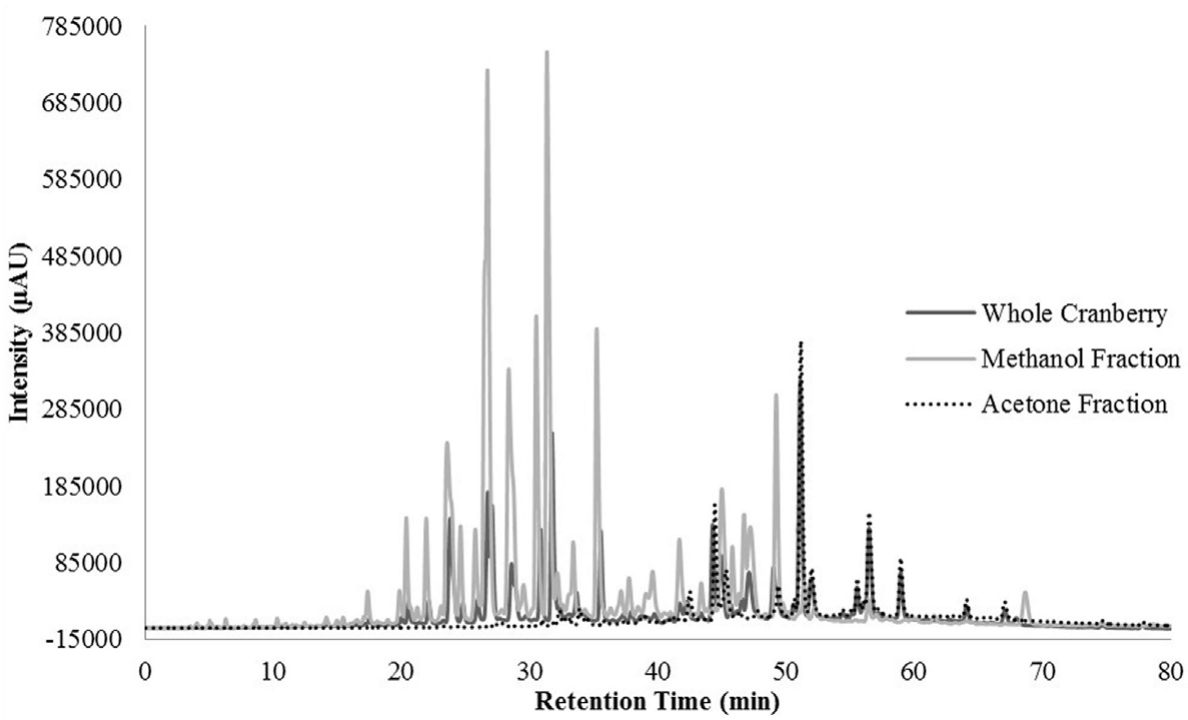
The chromatogram for the total scan from 200–600 nm for the whole cranberry (CR) extract and CR fractions.

**Figure 4:**
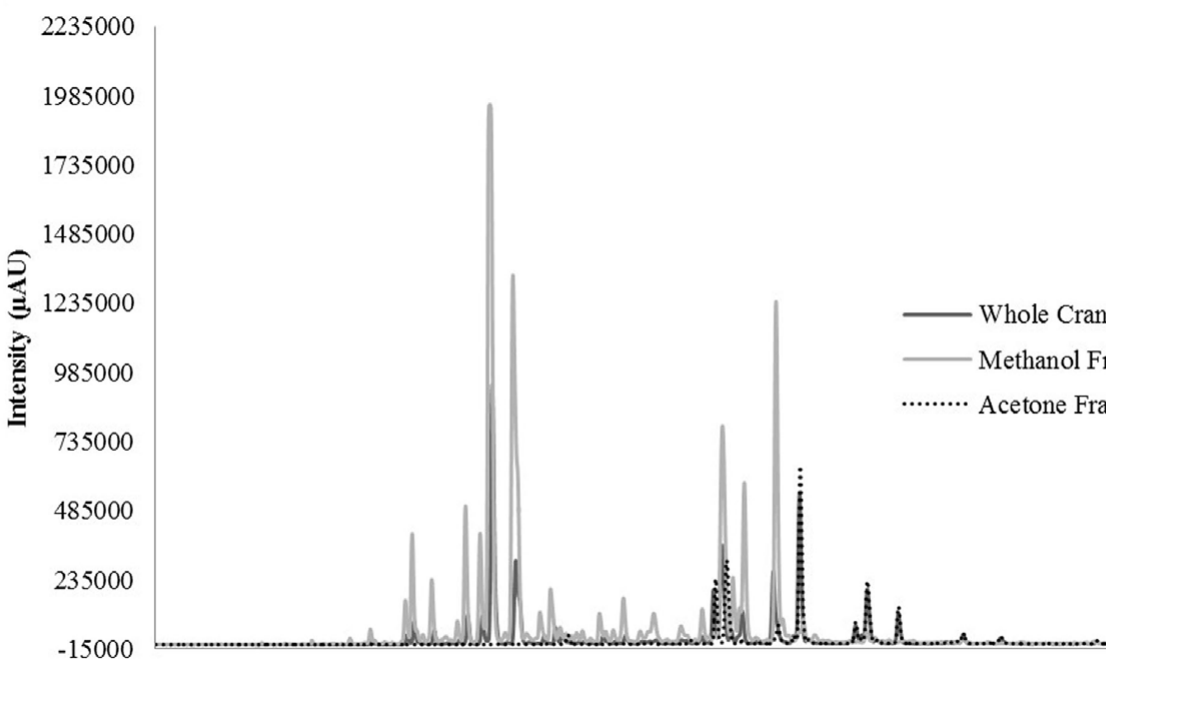
The chromatogram at 320 nm for the whole cranberry (CR) extract and CR fractions.

**Figure 5:**
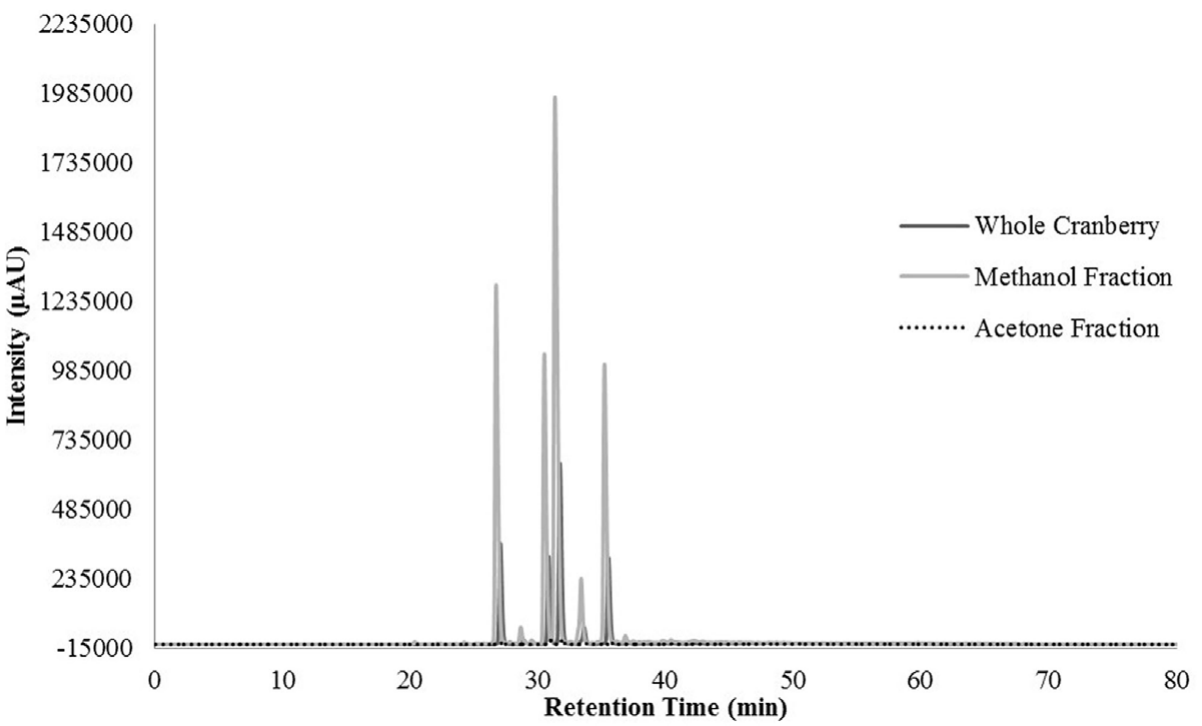
The chromatogram at 520 nm for the whole cranberry (CR) extract and CR fractions.

HPLC profiling was performed to understand the differences between the two tested CR fractions and to possibly identify phenolic compounds within the tested extracts. Unfortunately, we were unable to identify any compounds within our extracts, when compared to our existing library of compounds. However, we were able to identify differences between the two fractions that could confirm the effective fractionation. The total scan of the methanol fraction (CME) showed 10 major peaks with retention times between 24 and 49 min, which correspond to polar, low molecular weight phytochemicals present in cranberries (Figure 3). Seven of the peaks had a λ_max_ value of 320 nm (Figure 4), while the remaining peaks had a λ_max_ value of 520 nm (Figure 5). As expected, all anthocyanins that have λ_max_ value of 520 nm were eluted in the methanol fraction (Figure 5). The total scan of the acetone fraction had three major peaks with retention times after 50 min (Figure 3).

## Discussion

The GT extract powder had the highest TPC and free radical scavenging activity. This was expected since we used a standardized extract powder that contained 98% total phenols, 80% catechins, and 50% epigallocatechin gallate (EGCG). The CR extract powder contained 18% PACs. The TPC of the CR extract powder was less than half that of the GT extract and it needed to be diluted 10 times less than the GT extract. The TC extract powder had the lowest TPC and free radical scavenging activity. The TC extract powder contained 0.2% anthocyanins, in addition to maltodextrin, corn starch, and less than 0.25% lecithin, which was used as a processing aid.

All three extract powders tested showed α-glucosidase inhibition, suggesting a potential control of glucose production in the small intestine. When evaluated on the same phenolic content, the CR extract powder had the greatest inhibitory activity when compared to the GT and TC extract powders. This suggests that the phenolic compounds present in the tested CR extract are superior to GT and TC. Therefore, we suspect that the anthocyanins and PACs present in the CR extract were superior inhibitors of yeast α-glucosidase compared to the catechin (EGCG) present in the GT extract. Even though the most concentrated TC sample did not have more than 50% inhibition, the estimated IC_50_ value was about 50 times less than the GT extract powder. Similar to the CR extract powder, the anthocyanins present in the TC extract were able to inhibit yeast α-glucosidase better than the GT extract.

The bioactivity of catechins has been compared to anthocyanins and PACs in various studies. Seeram and Nair (22) determined the antioxidant activity of various catechins and anthocyanin/anthocyanidin compounds using fluorescence. In the study, it was determined that the anthocyanidin, delphinidin, had the greatest antioxidant activity with a value of 70.3 ± 1.1% (22). The most active catechin, (−)-catechin had an antioxidant activity of 65.4 ± 1.8% (22). The catechins and PACs from peanut skins were analyzed for their hypercholesterolemia suppressing activity in rats that were fed a high cholesterol diet (23). The PAC A1 from peanut skin inhibited the formation of micelles, where there was no micelle formation at a concentration of 1,250 µM (23). The (+)-catechin compound did not have an effect on the formation of micelles in rats (23). PACs and catechins extracted from *Ginkgo biloba* leaves were used to analyze the aggregation of amyloid β peptide and deposition of fibrils in the brain, which are regarded as key steps in the development of Alzheimer’s disease (24). PAC compounds, B1 and B3, had significantly lower IC_50_ values for the inhibition of the aggregation of amyloid β peptide than the catechins that were analyzed (24). The IC_50_ values of catechin, epicatechin (EC), gallocatechin, epigallocatechin, PAC B1, and PAC B3 were 14.93 ± 3.42, 9.44 ± 0.24, 17.54 ± 0.57, 8.51 ± 0.96, 3.28 ± 0.46, and 3.54 ± 0.39 µM, respectively (24). The PACs also had the highest destabilization activity of the preformed fibrils, but catechin and EC had similar results (24). The conclusion of the study was that PACs were better inhibitors of the aggregation of amyloid β peptides and initiators of the destabilization of the preformed fibrils, when compared to catechins (24). From the studies presented, it is evident that anthocyanins and PACs are more bioactive than catechins.

The CR extract powder was fractionated into two fractions, by using different polarity solvents. Initially, using 30% methanol we eluted higher polarity phenolic compounds, such as phenolic acids and anthocyanins. Later, by using 70% acetone we eluted the non-polar PACs. This method has been effectively used for many years for the separation of PACs, since Strumeyer and Malin (25) determined that tannins are adsorbed by Sephadex LH-20 resin in alcohol, but are released in acetone.

The total soluble phenolic content of CAE was higher than CME because the method measured the amount of phenolic functional groups within the sample. PAC molecules are composed of numerous phenolic ring structures, while anthocyanins only contain two phenolic rings. There could have been more anthocyanin molecules in CME overall, but the PAC molecules contributed more phenolic ring structures.

The antioxidant activity of CAE was higher than CME and the whole CR extract powder. CAE had a higher antioxidant activity than CME possibly due to the availability of the hydroxyl group proton donors in the extract. This was due to the molecular structure of anthocyanins and PACs. We suspect that PACs, which are predominantly present in CAE, have more hydrogen-donating hydroxyl groups than anthocyanins.

As stated in the results section, we believe that the phenolic phytochemicals in the methanol and acetone fractions must have a synergistic ability to inhibit α-glucosidase. CME also had better inhibitory activity of α-glucosidase because of the structure of the anthocyanin compounds. Since the interaction between kaempferol and α-glucosidase was dependent on hydrogen bonds and van der Waals forces, it can be hypothesized that the same criteria drives the interactions between PACs and/or anthocyanins and α-glucosidase (26). It is well defined that lower molecular weight phenolic compounds (such as phenolic acids and anthocyanins) and PACs have different mechanism of carbohydrate hydrolyzing enzyme inhibition. More specifically, it has been suggested that lower molecular weight phenolic compounds can act as specific inhibitors resulting to competitive or non-competitive inhibition (27, 28). On the other hand, larger molecular weight PACs have a very well-defined non-specific inhibition due to their well-defined protein-binding affinity (29). So we expect that anthocyanins and PACs are acting in synergy to inhibit α-glucosidase, since the observed α-glucosidase inhibitory activity of whole CR extract powder was greater than either of the two tested fractions.

The HPLC results indicated that CME contained more polar, low molecular weight phenolic phytochemicals and CAE contained less polar, high molecular weight phenolic phytochemicals. According to the literature, compounds with λ_max_ values of 320 nm were indicative of hydroxycinnamic acid type compounds and λ_max_ value of 520 nm corresponded to anthocyanin compounds (30). It should be noted that anthocyanins absorbed light at a higher wavelength because of the characteristic red color associated with anthocyanins (30). Therefore, CME contained hydroxycinnamic acid and other phenolic acids and anthocyanins.

Based on our findings, the CR-derived phenolic compounds had the greatest potential to control the glucose release, by the inhibition of α-glucosidase. The α-glucosidase inhibitory activity of the CR extract was due to the synergistic activity of the phenolic phytochemicals present in the tested extract. HPLC analysis determined that the methanol fraction contained the polar, low molecular weight phenolic phytochemicals, which included anthocyanins.

These findings are the first step for the development of a military ration with controlled glucose release. Further research is required for the determination of the specific phenolic phytochemicals present in the CR fractions. Better understanding of the mechanism of action will assist for the development of standardized, confirmatory animal and clinical trials.

## Author Contributions

AB executed all the experiments in the manuscript. DA and KR assisted in the execution of *in vitro* trials. SP directed the analytical experiments. EA designed the experiments and directed the research efforts.

## Conflict of Interest Statement

The authors declare that the research was conducted in the absence of any commercial or financial relationships that could be construed as a potential conflict of interest.
